# Risk of Incident Diabetes Mellitus Associated With the Dosage and Duration of Oral Glucocorticoid Therapy in Patients With Rheumatoid Arthritis

**DOI:** 10.1002/art.39537

**Published:** 2016-04-27

**Authors:** Mohammad Movahedi, Marie‐Eve Beauchamp, Michal Abrahamowicz, David W. Ray, Kaleb Michaud, Sofia Pedro, William G. Dixon

**Affiliations:** ^1^ Manchester Academic Health Science Centre and University of ManchesterManchesterUK; ^2^ McGill University Health CentreMontreal QuebecCanada; ^3^ McGill University Health Centre and McGill UniversityMontreal QuebecCanada; ^4^ National Data Bank for Rheumatic Diseases, Wichita, Kansas, and University of Nebraska Medical Center and Omaha VA Medical CenterOmaha; ^5^ National Data Bank for Rheumatic DiseasesWichitaKansas

## Abstract

**Objective:**

To quantify the risk of incident diabetes mellitus (DM) associated with the dosage, duration, and timing of glucocorticoid (GC) use in patients with rheumatoid arthritis (RA).

**Methods:**

We undertook a cohort study using 2 databases: a UK primary care database (the Clinical Practice Research Datalink [CPRD]) including 21,962 RA patients (1992–2009) and the US National Data Bank for Rheumatic Diseases (NDB) including 12,657 RA patients (1998–2013). Information on the dosage and timing of GC use was extracted. DM in the CPRD was defined using Read codes, at least 2 prescriptions for oral antidiabetic medication, or abnormal blood test results. DM in the NDB was defined through patient self‐reports. Data were analyzed using time‐dependent Cox models and a novel weighted cumulative dose (WCD) model that accounts for dosage, duration, and timing of treatment.

**Results:**

The hazard ratio (HR) was 1.30 (95% confidence interval [95% CI] 1.17–1.45) and 1.61 (95% CI 1.37–1.89) in current GC users compared to nonusers in the CPRD and the NDB, respectively. A range of conventional statistical models consistently confirmed increases in risk with the GC dosage and duration. The WCD model showed that recent GC use contributed the most to the current risk of DM, while doses taken >6 months previously did not influence current risk. In the CPRD, 5 mg of prednisolone equivalent dose for the last 1, 3, and 6 months was significantly associated with HRs of 1.20, 1.43, and 1.48, respectively, compared to nonusers.

**Conclusion:**

GC use is a clinically important and quantifiable risk factor for DM. Risk is influenced by the dosage and treatment duration, although only for GC use within the last 6 months.

Glucocorticoid (GC) therapy was first used to treat rheumatoid arthritis (RA) in 1948 and continues to be widely used in many inflammatory diseases. Two in three patients with RA have ever used GC therapy 1, reflecting the beneficial effects on symptom control and limitation of erosive disease progression 2, 3. However, there are concerns about a range of potential side effects 4, 5. Common side effects resulting from GC treatment are hyperglycemia and insulin resistance 6. Hyperglycemia results from GCs driving gluconeogenesis in the liver and antagonizing insulin‐mediated glucose disposal. However, there is much less clarity regarding whether and to what extent oral GC therapy leads to the development of diabetes mellitus (DM), a potentially irreversible event. Importantly, no studies consider the impact of dosage, duration, and timing of GC use and the risk of DM.

Many previous studies that quantify steroid side effects consider the relationship with current dosage (e.g., the risk with 5 mg or 10 mg prednisolone) but do not consider duration of use. Other models that consider long‐term exposure (e.g., ever use [7] or total cumulative dose [8,9]) are unable to account for changing patterns of GC exposure during follow‐up that may affect risk 10. For example, a patient “ever exposed” may have had GC therapy 5 years ago but not in the last 4 years, or alternatively, may still be actively receiving therapy. We have previously shown that a weighted cumulative dose (WCD) model that accounts for full exposure history predicted outcomes much better than conventional exposure models when examining the association between GC therapy and risk of infection 11. An added advantage of WCD modeling is that it generates a temporal relationship between drug exposure and the outcome of interest, allowing us to understand how risk relates to dosage, duration, and timing of therapy and enabling risk estimates for any given pattern of drug use.

The purpose of this study was to quantify the risk of incident DM in RA patients treated with GCs compared to RA patients not treated with GCs. Furthermore, we aimed to explore the relationship between dosage and timing of GC therapy and DM using conventional models and the novel WCD technique 12. The primary analysis was conducted using a primary care research database in the UK, and results were validated in a national US arthritis database.

## PATIENTS AND METHODS

### Clinical Practice Research Datalink (CPRD) (UK).

Patients with RA were identified from the CPRD, a database of anonymized UK primary care electronic medical records that is broadly representative of the UK population. The CPRD includes information for ∼11 million patients, generating more than 50 million person‐years of follow‐up 13. Information includes patient demographics, medical diagnoses, clinical test results, hospital referrals, and drug prescriptions.

In a retrospective cohort study design, patients with RA were identified from CPRD patients registered before October 2011. A validated algorithm 14 (with >80% sensitivity and specificity) was applied to identify adult patients with RA (for a list of Read codes, see Supplementary Table 1, available on the *Arthritis & Rheumatology* web site at http://onlinelibrary.wiley.com/doi/10.1002/art.39537/abstract). Patients age <16 years at the first RA code date were excluded. The study window was from January 1, 1992 to December 31, 2009. Patients with prevalent DM at study entry were excluded. Analysis was restricted to patients with >3 years of information in the CPRD prior to the first RA code date in order to assess prior GC exposure. Patients were followed up from the date of the first RA code within the study window until onset of DM, transfer out of general practice, last collection of general practice data, death, or December 31, 2009, whichever came first.

Oral GC therapy was identified from general practitioner prescriptions. Information on dosage and duration was extracted for each prescription, and dosages were converted into prednisolone equivalent dose (PED). In order to examine GC dosages typically seen in patients with RA, we excluded patients with at least 1 GC dose of >40 mg/day during their follow‐up, although we performed sensitivity analyses including these patients. Incident DM was defined as any one of the following: 1) a Read code for type 2 DM, 2) at least 2 prescriptions for oral antidiabetic medication (2 different medications or the same medication on 2 different dates), or 3) fasting blood sugar ≥7.0 mmoles/liter, random glucose level ≥11.1 mmoles/liter, glucose tolerance test result ≥11.1 mmoles/liter, or glycosylated hemoglobin (HbA_1c_) level ≥7%. The date of onset was considered the date of first recording of any of the above criteria within the study window. The study protocol was approved by the CPRD's Independent Scientific Advisory Committee (approval no. 11_113R).

### National Data Bank for Rheumatic Diseases (NDB) (US)

To validate results from the CPRD, we examined patients with RA participating in the NDB, a US longitudinal observational study described elsewhere 1, 15. Patients were recruited primarily from rheumatologists who confirmed the RA diagnosis, and were assessed semiannually with comprehensive questionnaires. Patients were required to complete at least 2 questionnaires between 1998 and 2013. Those who reported DM at enrollment were excluded. GC exposure was identified through self‐reported GC use in the preceding 6 months. We defined incident DM as occurring in the first month the patient reported treatment for DM or, if a new diagnosis but no treatment was reported, as occurring at a random time point within the 6‐month interval.

### Statistical analysis

The following a priori confounders were available in both settings and were adjusted for in the primary analysis: sex, age, history of hypertension, prior GC therapy in the 3 years preceding cohort entry, RA duration, concomitant time‐varying use of 4 disease‐modifying antirheumatic drugs (DMARDs) (methotrexate, hydroxychloroquine, sulfasalazine, and leflunomide), and use of nonsteroidal antiinflammatory drugs at any time during follow‐up. We also included family history of DM in the primary analysis for the CPRD.

Further sensitivity analyses were done in each data set, adjusting for additional confounders not available in the other setting. In the CPRD, analysis was additionally adjusted for cumulative dose of prior GCs in the 3 years preceding cohort entry, body mass index (BMI) at cohort entry, and smoking status at cohort entry. Missing data on BMI and smoking (22% and 4%, respectively) were handled using multiple imputations 16, 17. In the NDB, additional confounders included ethnicity, ever smoking status, BMI, employment status, rheumatic disease comorbidity index 18, total annual income, other DMARDs, use of biologic agents, and measures of disease severity (scores on the Health Assessment Questionnaire [HAQ] [19], pain scale, and global severity scale), all as time‐varying variables during follow‐up. Direct measures of disease activity were not available in either setting. However, no studies have yet shown an association between disease activity in RA and incident DM.

Primary analyses examined the association between GC exposure and time until incident DM, using multivariable Cox proportional hazards regression models 20 and their flexible WCD extension 12. Subjects who had not developed DM during follow‐up were right‐censored at the earliest of loss to follow‐up, end of the study period, or death. All models were adjusted for the same a priori confounders listed above. Age squared was added to the models to deal with nonlinearity, and disease duration was log‐transformed given its skewness. Effects of time‐varying GC exposure were estimated through adjusted hazard ratios (HRs) with 95% confidence intervals (95% CIs).

Because of uncertainty about mechanisms linking GC exposure and DM, we fitted 7 conventional models, each using a different representation of time‐varying GC exposure. Models 1 and 2 disregarded GC dosage and used binary time‐varying indicators of any past and/or current exposure until a given time point (model 1) or current use (model 2). Models 3 and 4 used current daily dose either as a continuous variable or as an ordinal variable, respectively, with cutoff points at 0, 5, 10, and 20 mg PED per day. Models 5 and 6 used continuous time‐varying measures of cumulative dose until a given time point, either in the last year or since study entry, respectively. Model 7 categorized cumulative dose since cohort entry, with cutoff points (based on quartiles) at 0, 960, 3,055, and 7,300 mg PED. Selected results were also presented as “number needed to harm,” i.e., the number of patients needed to be treated to cause an additional single case of DM 21.

The flexible WCD model represented time‐varying GC exposure as the weighted sum of past doses, with weights reflecting the relative importance of doses taken at different times in the past 12. Weights were estimated with cubic splines 12. Because of uncertainty regarding the time window over which past GC exposure may affect current DM risk and the complexity of the weight function, 4 alternative WCD models were fitted, with “exposure time windows” of 6 months and 1, 2, and 3 years and with different degrees of flexibility. For example, the model with the 1‐year window assumed that any dose taken ∼1 year ago or earlier had no impact on the current risk. All weight functions were a priori constrained to decay smoothly to zero at the end of the exposure time window, implying that drug doses taken at the corresponding time point had no impact on the current risk. Furthermore, for each window, we fitted 2 alternative models, with different flexibility and complexity, corresponding to 3df or 4df. The best‐fitting (lowest Akaike's information criterion [AIC] [22]) WCD model was initially selected in the CPRD 12, and its results were validated by comparison with the NDB estimates.

Goodness of fit of alternative models was compared with the minimum AIC, which provides an additional penalty for the increased complexity of WCD models, equivalent to an additional 2df for a posteriori choice of the best‐fitting “final” model among WCD models with alternative 1) exposure time windows and 2) df. Reduction of AIC by ≥10 points indicates an important improvement in the model's ability to predict outcomes 23 and identifies the model more likely to accurately represent the way past and current exposures affect risk 10.

## RESULTS

**Figure 1:**
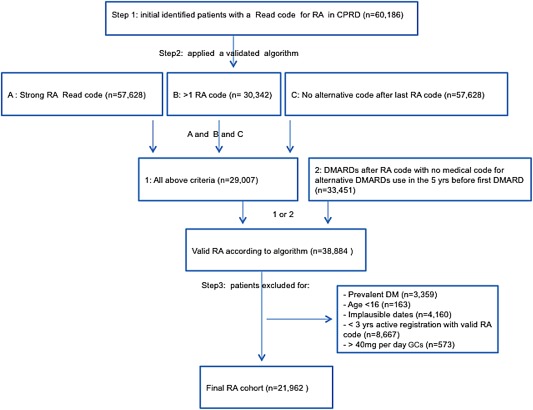
Clinical Practice Research Datalink (CPRD) rheumatoid arthritis (RA) cohort profile for patients with at least 3 years of active registration prior to a valid RA code. DMARDs = disease‐modifying antirheumatic drugs; DM = diabetes mellitus; GCs = glucocorticoids.

In the CPRD, among 60,186 subjects with any RA code, the validation algorithm identified 38,884 RA cases. Exclusion criteria reduced the cohort to 21,962 patients (Figure 1). Table 1 summarizes patient characteristics in the CPRD and the NDB and compares GC ever users to nonusers. Approximately 70% were female in both settings, and the proportion of users was similar in both settings. The mean age was 59 years in the CPRD and the NDB.

**Table 1:** Characteristics of populations in the CPRD and the NDB, stratified by oral GC therapy during follow‐up^a^

	CPRD			NDB		
	Total (n = 21,962)	Never used GC therapy (n = 12,066)	Ever used GC therapy (n = 9,896)	Total (n = 12,657)	Never used GC therapy (n = 6,658)	Ever used GC therapy (n = 5,999)
Female	70	71	70	80	80	80
Age at cohort entry, mean ± SD years	59 ± 15	58 ± 15	62 ± 14	59 ± 13	59 ± 14	59 ± 13
History of hypertension at baseline	23	22	24	44	42	45
Ever used NSAIDs during follow‐up	86	86	86	74	73	75
Ever used methotrexate during follow‐up	41	37	46	63	56	70
Ever used hydroxychloroquine during follow‐up	14	13	15	33	30	35
Ever used sulfasalazine during follow‐up	24	24	23	11	9	13
Ever used leflunomide during follow‐up	6	4	8	17	11	23
History of GC therapy in the 3 years before cohort entry	25	9	44	66	52	82
Percentage of time receiving GCs for those using GCs in the 3 years prior to cohort entry, mean ± SD	28 ± 31	11 ± 17	33 ± 31	26 ± 39	9 ± 22	47 ± 44
RA disease duration at cohort entry, mean ± SD years	2 ± 3	2 ± 3	2 ± 3	14 ± 11	13 ± 12	14 ± 11
Family history of DM	13	14	12	–	–	–
BMI, mean ± SD kg/m^2^ b	27 ± 6	27 ± 6	26 ± 6	28 ± 7	28 ± 7	28 ± 7
Missing BMI data	22	22	21	–	–	–
Smoking status at cohort entry						
In NDB (ever)	–	–	–	47	49	45
In CPRD						
Nonsmoker	47	48	46	–	–	–
Former smoker	17	16	17	–	–	–
Current smoker	32	32	32	–	–	–
Missing data	4	4	5	–	–	–
HAQ score during follow‐up, mean ± SD (range 0–3)	–	–	–	1 ± 1	1 ± 1	1 ± 1
Global severity during follow‐up, mean ± SD (0–10‐cm VAS)	–	–	–	4 ± 2	3 ± 2	4 ± 2
Employed during follow‐up	–	–	–	31	34	27
Total annual income during follow‐up, mean ± SD US dollars	–	–	–	50,418 ± 30,140	52,060 ± 30,487	48,595 ± 29,654
Rheumatic disease comorbidity index during follow‐up, mean ± SD (range 0–9)	–	–	–	1.7 ± 1.3	1.5 ± 1.3	1.8 ± 1.3
Biologic agents during follow‐up	–	–	–	47	39	55
Other DMARDs during follow‐up	–	–	–	7	5	9

a Except where indicated otherwise, values are the percent of patients. GC = glucocorticoid; NSAIDs = nonsteroidal antiinflammatory drugs; RA = rheumatoid arthritis; DM = diabetes mellitus; BMI = body mass index; HAQ = Health Assessment Questionnaire; VAS = visual analog scale; DMARDs = disease‐modifying antirheumatic drugs.

b At baseline in the Clinical Practice Research Datalink (CPRD) and average throughout the follow‐up period in the National Data Bank for Rheumatic Diseases (NDB).

In the CPRD, 9,896 patients (45%) received at least 1 prescription for oral GCs. Of these patients, 6,886 (70%) started their follow‐up unexposed. GC users were on average 4 years older than nonusers.

Patients who received GC therapy during follow‐up were more likely than nonusers to have received GCs in the 3 years prior to the first RA code (44% versus 9%). Methotrexate, hydroxychloroquine, and leflunomide, but not sulfasalazine, were prescribed in a higher proportion of GC users during follow‐up. There were no marked differences in BMI, smoking status, hypertension, or family history of DM between GC users and nonusers (Table 1).

In the NDB, 5,999 patients (47%) were ever exposed to oral GCs during follow‐up. Of these patients, 2,344 (39%) started their follow‐up unexposed. Similar to patients in the CPRD, patients in the NDB who ever received GC therapy had higher frequencies of DMARD use and higher prior GC use. Patients in the NDB who ever received GC therapy also had a higher frequency of biologic agent use, which was not captured in the CPRD. They also had worse RA severity, more comorbidities, and higher unemployment (Table 1). During periods of GC use, the median daily dose was 6.5 mg PED (interquartile range [IQR] 4.0–7.5 mg) in the CPRD and 6.0 mg PED (IQR 4.0–7.0 mg) in the NDB.

In the CPRD, 2,260 patients (10%) were diagnosed as having new‐onset DM during a median follow‐up period of 5.4 years, giving an incidence of 16.7 per 1,000 person‐years (Table 2). More than half of DM cases (57%) were first identified through abnormal blood test results, 36% through DM Read codes, and 3% through antidiabetic medication. A total of 1,209 cases were identified in patients not yet exposed to GC therapy (incidence of 13.9 per 1,000 person‐years), and 1,051 cases were identified in those ever exposed (incidence of 21.8 per 1,000 person‐years). In the NDB, 861 patients (6.8%) were diagnosed as having DM during a median follow‐up period of 3.4 years (incidence of 14.2 per 1,000 person‐years). The incidences for those not yet exposed and those ever exposed were 12.0 per 1,000 person‐years and 16.9 per 1,000 person‐years, respectively.

**Table 2:** Observation time and incidence of DM by oral GC status^a^

	CPRD			NDB		
	All (n = 21,962)	Not yet used GC therapy (n = 18,942)^b^	Ever used GC therapy (n = 9,896)	All (n = 12,657)	Not yet used GC therapy (n = 9,002)†	Ever used GC therapy (n = 5,999)
Total person‐years	135,007	86,706	48,300	60,544	33,433	27,111
Incident cases of DM	2,260	1,209	1,051	861	402	459
Time at risk per subject, median years	5.40	3.38	3.91	3.42	2.42	3.42
Incidence per 1,000 person‐years (95% CI)	16.7 (16.1–17.4)	13.9 (13.2–14.8)	21.8 (20.5–23.1)	14.2 (13.3–15.2)	12.0 (10.9–13.3)	16.9 (15.4–18.6)

a DM = diabetes mellitus; CPRD = Clinical Practice Research Datalink; NDB = National Data Bank for Rheumatic Diseases; 95% CI = 95% confidence interval.

b Patients could contribute person‐time to the group that had not yet used glucocorticoid (GC) therapy and then switch to person‐time in the group that had ever used GC therapy on receipt of their first prescription for GCs.

Table 3 shows the associations between oral GC use and risk of DM, estimated through alternative Cox models. All models consistently suggested in both data sets that DM risk was associated with GC use and increased with higher dosage and/or longer treatment duration. After adjustment for comparable confounders, the HR for those who were ever exposed compared with those who were never exposed (model 1) was 1.35 (95% CI 1.22–1.48) and 1.42 (95% CI 1.22–1.66) in the CPRD and the NDB, respectively. This equates to 1 additional case of DM per year for every 206 and 158 patients ever receiving GCs in the CPRD and the NDB, respectively. There was a similar increased risk of DM associated with current use of oral GCs (model 2) in the CPRD (HR 1.30 [95% CI 1.17–1.45]) and the NDB (HR 1.61 [95% CI 1.37–1.89]). Each 5 mg increase of current oral GC dosage was associated with a 25% and 30% increased risk of DM in the CPRD and the NDB, respectively (model 3).

**Table 3:** Association between DM incidence and oral GC exposure for 7 conventional models and the best‐fitting WCD model^a^

		CPRD		NDB	
Model no.	Model description	HR (95% CI)^b^	AIC	HR (95% CI)†	AIC
1	Ever used (reference: not yet used)	1.35 (1.22–1.48)	41,277.6	1.42 (1.22–1.66)	14,616.8
2	Current user (reference: nonuser)	1.30 (1.17–1.45)	41,293.1	1.61 (1.37–1.89)	14,604.3
3	Current dosage (5 mg/day)	1.25 (1.19–1.31)	41,250.0	1.30 (1.21–1.38)	14,590.4
4	Current dosage, category (mg/day)		41,256.5		14,587.5
	None	1.00		1.00	
	Low (0–4.9)	1.06 (0.87–1.28)		1.07 (0.80–1.40)	
	Medium (5–9.9)	1.16 (1.00–1.34)		1.58 (1.30–1.93)	
	High (10–19.9)	1.97 (1.61–2.40)		2.24 (1.72–2.93)	
	Very high (≥20)	3.19 (2.22–4.58)		3.06 (1.90–4.91)	
5	Cumulative dose in the last year (per 1,000 mg increase)	1.22 (1.17–1.28)	41,252.5	1.19 (1.14–1.24)	14,589.2‡
6	Cumulative dose since cohort entry (per 1,000 mg increase)	1.02 (1.01–1.03)	41,300.7	1.03 (1.02–1.05)	14,613.2
7	Cumulative dose since cohort entry, mg		41,278.3		14,614.6
	None	1.00		1.00	
	Low (0–959.9)	1.23 (1.08–1.40)		1.21 (0.97–1.51)	
	Medium (960–3,054.9)	1.41 (1.22–1.62)		1.36 (1.08–1.70)	
	High (3,055–7,298.9)	1.35 (1.15–1.57)		1.68 (1.35–2.11)	
	Very high (≥7,299)	1.53 (1.30–1.79)		1.67 (1.31–2.12)	
8	Final best selected fit WCD model (12 months, 3df for both data sets)	HR varies according to pattern of GC use and reference (see Table 4 for details)	41,232.7^c^	HR varies according to pattern of GC use and reference (see Table 4 for details)	14,589.6

a WCD = weighted cumulative dose; NDB = National Data Bank for Rheumatic Diseases; HR = hazard ratio; 95% CI = 95% confidence interval; AIC = Akaike's information criterion.

b Adjusted for comparable confounders in both data sets (sex, age, history of hypertension, ever use of nonsteroidal antiinflammatory drugs at cohort entry, concomitant time‐varying use during follow‐up of 4 main disease‐modifying antirheumatic drugs, duration of rheumatoid arthritis, and use of glucocorticoids [GCs] in the 3 years prior to cohort entry); additionally adjusted for family history of diabetes mellitus (DM) in the Clinical Practice Research Datalink (CPRD).

c Best‐fitting model in each data set.

Among the confounders, hydroxychloroquine use was consistently associated with lower incident risk of DM in both the CPRD and the NDB (HRs of 0.79 [95% CI 0.64–0.99] and 0.64 [95% CI 0.53–0.76], respectively) (see Supplementary Table 2, available on the *Arthritis & Rheumatology* web site at http://onlinelibrary.wiley.com/doi/10.1002/art.39537/abstract). A history of hypertension at baseline was associated with higher DM incidence in both the CPRD and the NDB (HRs of 1.86 [95% CI 1.70–2.05] and 1.78 [95% CI 1.55–2.05], respectively). Comparison of AIC values indicated that in both settings the models’ fit was substantially improved if current dosage or cumulative dose in the last year was taken into account (Table 3). There was no marked change in risk estimates for current dosage (5 mg/day) (model 3) in sensitivity analyses done, in both settings, either by adjusting for additional confounders or by including patients with prescriptions of >40 mg PED (see Supplementary Tables 3 and 4, available on the *Arthritis & Rheumatology* web site at http://onlinelibrary.wiley.com/doi/10.1002/art.39537/abstract).

**Figure 2:**
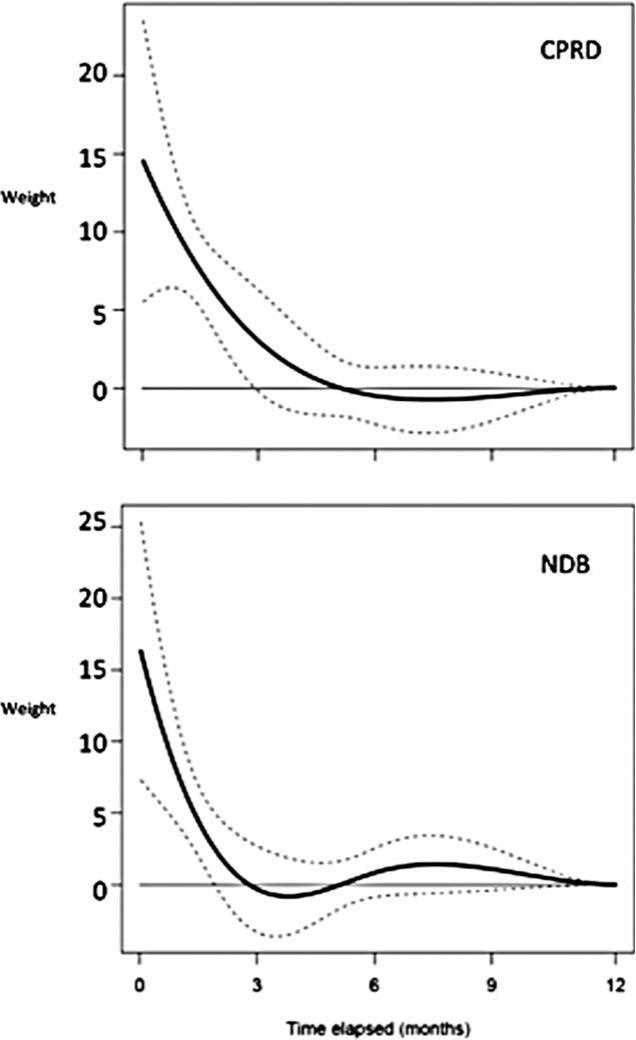
Estimated weight function (solid curve) from the best‐fitting weighted cumulative dose model, with 95% confidence limits (dashed curves), in the Clinical Practice Research Datalink (CPRD) and the National Data Bank for Rheumatic Diseases (NDB). Weight functions are 1/1,000 of the stated values (e.g., weight of 10 = 0.0010). The higher weight function in the most recent time period suggests that more recent treatment has a greater impact on risk of diabetes mellitus, while the absent weight function beyond 6 months suggests that exposures more than 6 months ago have no impact on the current risk of diabetes mellitus.

In the CPRD, the WCD model fitted data substantially better than any conventional models, with at least 17 points improvement in AIC (Table 3). The weight function estimated through the best‐fitting WCD model, with a 12‐month window, indicates that risk of DM increases with increasing cumulative dose in the most recent few months (Figure 2). Doses taken in the last few weeks have the highest impact, while those taken >5 months ago do not materially affect the risk, as indicated by all weights assigned to elapsed times >5 months being very close to zero (Figure 2). In the NDB, current dosage, cumulative dose in the last year, and WCD (models 3, 5, and 8) fit the data similarly well, with AIC differences of <2 points (Table 3). The NDB 12‐month WCD weight function confirmed that recent doses have the strongest impact, with minimal effect of doses taken >6 months ago. For both settings, WCD models with longer time windows found similar weight functions, confirming no effect of doses taken >1 year ago (see Supplementary Figure 1, available on the *Arthritis & Rheumatology* web site at http://onlinelibrary.wiley.com/doi/10.1002/art.39537/abstract).

Table 4 shows the adjusted HRs for DM associated with different clinically plausible patterns of GC therapy, estimated using the best‐fitting 12‐month CPRD WCD model. Compared to nonuse, taking 5 mg PED for the last 1, 3, and 6 months was significantly associated with 20%, 43%, and 48% increases, respectively, in risk. The fact that extending duration of past exposure beyond 3 months had only a very minor impact on the estimated hazard of DM occurrence reflects the very low weights estimated for GC exposures that occurred >3–4 months ago (Figure 2). On the other hand, risks associated with cumulative exposure at a high daily dose of 30 mg PED were much higher, with 1, 3, and 6 months of past use associated with 3–10‐fold increases in the risk of DM (Table 4). Accordingly, 5 mg or 30 mg PED for 6 months should add 1 case of DM for every 133 or 7 patients treated, respectively. Past users who discontinued GC therapy at least 6 months ago had a risk of DM comparable to that of those who never used GCs (Table 4).

**Table 4:** Adjusted HRs for association between risk of DM incidence and selected clinical pattern of GC therapy using Clinical Practice Research Datalink data from the best‐fitting weighted cumulative dose model (12 months, 3df)^a^

Pattern of use of GCs	Reference	Adjusted HR (95% CI)^b^
Current user, 5 mg/day for last 1 month	Nonuser	1.20 (1.11–1.29)
Current user, 5 mg/day for last 3 months	Nonuser	1.43 (1.29–1.57)
Current user, 5 mg/day for last 6 months	Nonuser	1.48 (1.33–1.64)
Current user, 5 mg/day for last 1 year	Nonuser	1.42 (1.30–1.54)
Current user, 5 mg/day for last 3 years	Nonuser	1.42 (1.30–1.54)
Past user, 5 mg/day for 6 months, stopped 3 months ago	Nonuser	1.00 (0.90–1.12)
Past user, 5 mg/day for 6 months, stopped 6 months ago	Nonuser	0.96 (0.86–1.07)
Current user, 5 mg/day for last 6 months	Past user, 5 mg/day for 6 months, stopped 3 months ago	1.47 (1.25–1.73)
Current user, 30 mg/day for last 1 month	Nonuser	2.93 (1.83–4.61)
Current user, 30 mg/day for last 3 months	Nonuser	8.41 (4.71–15.0)
Current user, 30 mg/day for last 6 months	Nonuser	10.4 (5.54–19.4)
Current user, 30 mg/day for last 1 month	Past user, 30 mg/day for 1 month, stopped 1 month ago	1.49 (0.97–2.29)
Current user, 30 mg/day for last 1 month	Past user, 30 mg/day for 1 month, stopped 3 months ago	2.44 (1.26–4.75)

a HR = hazard ratio; 95% CI = 95% confidence interval.

b Adjusted for sex, age, history of hypertension, ever use of nonsteroidal antiinflammatory drugs at cohort entry, duration of rheumatoid arthritis, use of glucocorticoids [GCs] in the 3 years prior to cohort entry, family history of diabetes mellitus (DM), and concomitant time‐varying use during follow‐up of 4 main disease‐modifying antirheumatic drugs.

## DISCUSSION

It is accepted that oral GC therapy is an important risk factor for DM. However, to date this risk has not been well quantified, and no studies have considered the impact of dosage, duration, *and* timing of GC use on risk of DM. Using 2 distinct data sets of patients with RA, we have generated validated measures of risk of DM for various patterns of GC use. Risk increases with dosage—each 5 mg increase of current oral GCs was associated with a 25–30% increased risk of DM. We have also shown that only those GC doses taken within the preceding 6 months are associated with current risk of DM. The use of 2 data sets with distinct study designs and geographic settings adds significant validity to the findings. Despite different populations, methods of ascertaining DM, and definitions of exposure, the incidence of DM was nearly identical in the 2 studies, as were estimates of risk for different models of GC therapy.

Existing literature on the association between GC therapy and risk of DM can appear inconsistent and at times conflicting, with studies showing increased risk 7, 24, no association 7, 24, 25, and even lower incidence with cumulative dose 8, 9. Our analyses consistently showed an association between DM and GC therapy, increasing with higher dosage and/or longer treatment duration. This is consistent with previous studies showing positive associations and high DM incidence with higher dosages of prednisolone 7, 24, 26. Findings in the studies by Wasko et al 8 and by Di Comite and Rossi 9, which showed increasing cumulative prednisolone exposure to be associated with a *lower* incidence of DM, conflict with findings from both the CPRD and the NDB. The reason for the discrepancy is not clear as similar adjustments were made. However, reduced risk with increasing cumulative exposure does not seem biologically plausible.

The RA patient population in the CPRD was identified using a validated algorithm. The sensitivity and specificity of the algorithm (84% and 86%, respectively) mean that there would have been some misclassification which would have likely diluted the strength of the estimated association 27. However, replication of our CPRD findings in a second cohort with similar magnitudes of risk adds weight to our findings and provides strong reassurance that the results are both accurate and generalizable to other RA patient populations. We found in both cohorts that current use of GCs at <5 mg PED per day was not associated with a significantly increased risk of DM, perhaps suggesting a dosage with a more favorable balance of benefit and harm.

Although not the primary focus of this study, it was interesting to note the HRs for DMARD therapy. There was a consistent protective association of hydroxychloroquine with DM (HRs of 0.79 [95% CI 0.64–0.99] and 0.64 [95% CI 0.53–0.76] in the CPRD and the NDB, respectively). The protective effect of hydroxychloroquine on incidence of DM has been suggested in previous studies in RA 8, 28, 29. If disease severity is associated with the risk of incident DM, this may be partially explained by confounding, as hydroxychloroquine is prescribed preferentially for patients with less severe disease. Nonetheless, chloroquines are known to suppress plasma glucose, even inducing hypoglycemia. The mechanism of action remains unclear, but increased insulin production and enhanced insulin action both appear to play roles 30. The associations with leflunomide appeared different in the 2 cohorts, perhaps reflecting different patterns of use of this DMARD between the 2 countries.

Conventional analysis models make imperfect assumptions about the importance of the timing of medication use with respect to the outcome. For example, current dosage models ignore the impact of any historical therapy, while cumulative exposure assumes that doses taken years ago have the same importance as doses taken recently. The WCD model avoids such assumptions, allowing the data to define the importance of dosage according to its recency. The output of the WCD analysis is twofold. First, it allows us to estimate risks for any given pattern of GC exposure; providing risk estimates for clinically plausible patterns of GC use allows clinicians and patients to make more informed decisions. Second, the shape of the WCD curves in both data sets suggests that only those doses within the last 6 months are associated significantly with current risk of DM. This suggests that the acute metabolic effects of GC exposure are dominant, but also that GC‐associated glucose intolerance, and thereby risk of DM, is reversible.

As in all observational studies, the impact of bias, including confounding, needs to be considered. In particular, unmeasured confounding could bias our results if some variable(s) associated with both the exposure (GC use) and the outcome (DM) was not recorded in the study databases and thus not adjusted for in the analysis. Confounding by indication can be a major challenge in observational drug studies if the indication for treatment (in our case RA disease severity) is associated with the outcome 31.

We have been unable to find any published evidence of disease severity in RA being associated with incident DM. Indeed, some studies have found RA disease severity not to be associated with progression to impaired glucose tolerance or incident DM 26; thus, confounding by indication (or confounding by disease severity) is unlikely. The association between disease severity and other measures of glucose metabolism appears inconclusive, with several studies showing no association 25, 32, 33, 34 but other studies showing positive associations 35, 36. Despite this uncertainty, we adjusted for a range of possible confounders in both data sets, including smoking, BMI, and time‐varying exposure to DMARDs. Adjustment for smoking and BMI has previously been shown to attenuate the association between RA and DM 37. Although there was no direct measurement of disease activity or biologic therapy in the CPRD, it was reassuring that the risk remained significantly increased after additional adjustment for both HAQ score and current use of biologic agents in our analyses of the NDB. However, we cannot exclude a risk of confounding by some other unmeasured characteristics associated with both GC treatment (and its intensity) and incident DM.

One possible bias that could not be controlled for is surveillance bias. As acknowledged in the introduction, the association between GC therapy and incident DM is well known although poorly quantified. This might mean that clinicians have a greater tendency to test patients for new‐onset DM while they are receiving steroids. We examined the frequency with which blood glucose was measured in patients exposed and those not exposed to GCs in the CPRD, and we found that the frequency was 406 per 1,000 patient‐years in those ever exposed to GCs and 289 per 1,000 patient‐years in those never exposed to GCs. While this might initially look like a surveillance bias, any true increased incidence of DM will lead to symptoms in the GC‐treated patients, consultation with their doctors, and an appropriate test (and positive test result) for DM. Thus, the increased frequency of tests will be a combination of possible surveillance bias and testing secondary to symptoms. Unfortunately, it is not possible to ascertain within the CPRD whether blood tests are screening tests or are performed because of clinical suspicion.

The results of this study have several applications. Most importantly, the quantification of the risk of DM with various dosages and durations of GC therapy allows clinicians and patients to make informed decisions about their treatment, balancing benefits and harms as is advocated in European guidelines 38. Having quantified the risk of DM for any pattern of steroid use, we plan further work to examine the threshold for cost‐effective DM screening in patients receiving steroids for RA. By using novel analytic methods with replication in a second data set, we can be increasingly confident about the quantified risks of DM conferred by this commonly used drug.

## AUTHOR CONTRIBUTIONS

All authors were involved in drafting the article or revising it critically for important intellectual content, and all authors approved the final version to be published. Dr. Dixon had full access to all of the data in the study and takes responsibility for the integrity of the data and the accuracy of the data analysis.

**Study conception and design.** Movahedi, Abrahamowicz, Ray, Michaud, Dixon.

**Acquisition of data.** Movahedi, Michaud, Pedro, Dixon.

**Analysis and interpretation of data.** Movahedi, Beauchamp, Abrahamowicz, Ray, Michaud, Pedro, Dixon.

## Supporting information

Supplementary Table 1. Read code list to define RA
Supplementary Table 2. The association between comparable confounders and risk of Type II DM in the two datasets
Supplementary Table 3. Sensitivity analyses using Model 3 in CPRD
Supplementary Table 4. Sensitivity analyses using Model 3 in NDB
Supplementary Figure 1. WCD models with all time windows tested (3 degrees of freedom)
