# Supplementary material for: Risk of Incident Diabetes Mellitus Associated With the Dosage and Duration of Oral Glucocorticoid Therapy in Patients With Rheumatoid Arthritis
Source: Arthritis Rheumatol. 2016 Apr 27;68(5):1089–98. doi: 10.1002/art.39537 (PMC4982029; doi:10.1002/art.39537)
Supplement: Supplementary file 1 — Supplementary Table 1. Read code list to define RA Supplementary Table 2. The association between comparable confounders and risk of Type II DM in the two datasets Supplementary Table 3. Sensitivity analyses using Model 3 in CPRD Supplementary Table 4. Sensitivity analyses using Model 3 in NDB Supplementary Figure 1. WCD models with all time windows tested (3 degrees of freedom) [file ART-68-1089-s001.doc]

**Supplementary materials**

- **Definition of RA using validated algorithm**
- **Supplementary Table 1. Read code list to define RA**
- **Supplementary Figure 1. WCD models with all time windows tested (3 degrees of freedom)**
- **Supplementary Table 2. The association between comparable confounders and risk of Type II DM in two dataset**
- **Sensitivity analysis using Model 3 (current dose; 5mg/day)**
- Supplementary Table 3. Sensitivity analyses using Model 3 in CPRD
- Supplementary Table 4. Sensitivity analyses using Model 3 in NDB

**Definition of RA**

The following steps were followed to define the RA cohort in CPRD:

Step 1. Any patient with a Read code for RA was initially identified (see code list for RA below).

Step 2. A validated algorithm was then applied (1), restricting RA patients to those who had either:

- 1. All of the following criteria (A & B & C)

A. At least one RA code categorized as seropositive/erosive RA or “rheumatoid arthritis” code (RA groups 1 or 2 in code list below), *and*

1. More than one RA code on different days, *and*
2. No alternative diagnostic code after last RA code

OR

- 1. DMARD prescription and no medical code for an alternative indication for the specific DMARD in the 5 years before the first prescription date,

The date of first RA code was defined as the first ever date on which the above criteria were met.

**Supplementary Table 1. Read** code list to define RA

|  | **Read code** | **Description** | **RA group*** |
| --- | --- | --- | --- |
| 1 | Nyu1G00 | [X]SEROPOSITIVE RHEUMATOID ARTHRITIS, UNSPECIFIED | 1 |
| 2 | N047.00 | SEROPOSITIVE ERROSIVE RHEUMATOID ARTHRITIS | 1 |
| 3 | N04X.00 | SEROPOSITIVE RHEUMATOID ARTHRITIS, UNSPECIFIED | 1 |
| 4 | Nyu1100 | [X]OTHER SEROPOSOTIVE RHEUMATOID ARTHRITIS | 1 |
| 5 | N04y200 | ADULT-ONSET STILL'S DISEASE | 2 |
| 6 | N040T00 | FLARE OF RHEUMATOID ARTHRITIS | 2 |
| 7 | 14G1.00 | H/O: RHEUMATOID ARTHRITIS | 2 |
| 8 | N040100 | OTHER RHEUMATOID ARTHRITIS OF SPINE | 2 |
| 9 | 66H..13 | RHEUMATOID ARTHRIT. MONITORING | 2 |
| 10 | N040.00 | RHEUMATOID ARTHRITIS | 2 |
| 11 | N040S00 | RHEUMATOID ARTHRITIS - MULTIPLE JOINT | 2 |
| 12 | N040F00 | RHEUMATOID ARTHRITIS OF ANKLE | 2 |
| 13 | N040000 | RHEUMATOID ARTHRITIS OF CERVICAL SPINE | 2 |
| 14 | N040600 | RHEUMATOID ARTHRITIS OF DISTAL RADIO-ULNAR JOINT | 2 |
| 15 | N040500 | RHEUMATOID ARTHRITIS OF ELBOW | 2 |
| 16 | N040B00 | RHEUMATOID ARTHRITIS OF HIP | 2 |
| 17 | N040D00 | RHEUMATOID ARTHRITIS OF KNEE | 2 |
| 18 | N040800 | RHEUMATOID ARTHRITIS OF MCP JOINT | 2 |
| 19 | N040900 | RHEUMATOID ARTHRITIS OF PIP JOINT OF FINGER | 2 |
| 20 | N040200 | RHEUMATOID ARTHRITIS OF SHOULDER | 2 |
| 21 | N040700 | RHEUMATOID ARTHRITIS OF WRIST | 2 |
| 22 | N005.00 | ADULT STILL'S DISEASE | 2 |
| 23 | N040A00 | RHEUMATOIOD ARTHERITIS OF DIP JOINT OF FINGER | 2 |
| 24 | Nyu1200 | [X]OTHER SPECIFIED RHEUMATOID ARTHRITIS | 2 |
| 25 | N040H00 | RHEUMATOIOD ARTHERITIS OF TALONAVICULAR JOINT | 2 |
| 26 | N040J00 | RHEUMATOIOD ARTHERITIS OF OTHER TARSAL JOINT | 2 |
| 27 | N040G00 | RHEUMATOIOD ARTHERITIS OF SUBTALAR JOINT | 2 |
| 28 | N040L00 | RHEUMATOIOD ARTHERITIS OF LESSER MTP JOINT | 2 |
| 29 | 38DZ.00 | DISEASE SEVERITY SCORE IN RHEUATOID ARTHRITIS | 2 |
| 30 | N040C00 | RHEUMATOIOD ARTHERITIS OF SACRO-ILLIAC JOINT | 2 |
| 31 | N040400 | RHEUMATOIOD ARTHERITIS OF ACROMIOCLAVICULAR JOINT | 2 |
| 32 | N040K00 | RHEUMATOID ARTHRITIS OF FIRSTH MTP JOINT | 2 |
|  | **Read code** | **Description** | **RA group*** |
| 33 | N042200 | RHEUMATOID NODULE | 3 |
| 34 | N040N00 | RHEUMATOID VASCULITIS | 3 |
| 35 | G5yA.00 | RHEUMATOID CARDITIS | 3 |
| 36 | H570.00 | RHEUMATOID LUNG | 3 |
| 37 | N042100 | RHEUMATOID LUNG DISEASE | 3 |
| 38 | G5y8.00 | RHEUMATOID MYOCARDITIS | 3 |
| 39 | N040R00 | RHEUMATOID NODULE | 3 |
| 40 | N04y011 | CAPLAN’S SYNDROME | 3 |
| 41 | N041.00 | FELTY'S SYNDROME | 3 |
| 42 | N04y012 | FIBROSING ALVEOLITIS ASSOCIATED WITH RHEUMATOID ARTHRITIS | 3 |
| 43 | F396400 | MYOPATHY DUE TO RHEUMATOID ARTHRITIS | 3 |
| 44 | F371200 | POLYNEUROPATHY IN RHEUMATOID ARTHRITIS | 3 |
| 45 | N042.00 | OTHER RHEUMATOID ARTHROPATHY+VISCERAL/SYSTEMIC INVOLVEMENT | 3 |
| 46 | N042z00 | RHEUMATOID ARTHROPATHY + VISCERAL/SYSTEMIC INVOLVEMENT NOS | 3 |
| 47 | N04y000 | RHEUMATOID LUNG | 3 |
| 48 | N04..00 | RHEUMATOID ARTHRITIS & OTHER INFLAMMATORY POLYARTHROPATHY | 4 |
| 49 | N040P00 | SERONEGATIVE RHEUMATOID ARTHRITIS | 4 |
| 50 | 7P20300 | DELIEVERY OF REHABILITATION FOR RHEUMATOID ARTHRITIS | 4 |

* Group 1: Sero+ve/ erosive, group 2: RA, group 3, extraarticular manifestations, group 4: sero-ve RA/ other weak evidence. ‘Strong codes’ are those in Groups 1 and 2.

**Supplementary Figure 1. WCD models with all time windows tested (3 degrees of freedom)**

**Supplementary Table 2. The association between comparable confounders and risk of Type II DM in the two datasets**

|  |  | CPRD | | NDB | |
| --- | --- | --- | --- | --- | --- |
|  |  | WCD, 12 month, 3 df*  HR (95% CI) | Current dose  (Model 3)  HR (95% CI) | WCD, 12 month, 3 df*  HR (95% CI) | Current dose  (Model 3)  HR (95% CI) |
| 1 | Gender (male) | 1.34 (1.23 - 1.46) | 1.35 (1.24 - 1.48) | 1.00 (0.85 - 1.19) | 1.01 (0.85 - 1.19) |
| 2 | Age at cohort entry (HR per 10 years increase) | 2.27 (1.76 - 2.92) | 2.27 (1.78 - 2.90) | 2.03 (1.31 - 3.13) | 2.02 (1.30 - 3.13) |
| 3 | Age at cohort entry squared (HR per 100 years increase) | 0.94 (0.92 - 0.96) | 0.94 (0.92 - 0.96) | 0.94 (0.90 - 0.97) | 0.94 (0.90 - 0.97) |
| 4 | Family history of DM | 1.46 (1.31 - 1.63) | 1.46 (1.31 - 1.62) | - | - |
| 5 | History of hypertension before cohort entry | 1.86 (1.69 - 2.05) | 1.86 (1.70 - 2.05) | 1.78 (1.55 - 2.05) | 1.78 (1.55 - 2.05) |
| 6 | Ever used NSAID during follow-up | 1.06 (0.95 - 1.19) | 1.06 (0.94 - 1.19) | 0.75 (0.64 - 0.89) | 0.74 (0.63 - 0.88) |
| 7 | Methotrexate use during follow-up | 0.98 (0.89 - 1.09) | 0.98 (0.89 - 1.09) | 0.73 (0.63 - 0.83) | 0.73 (0.63 - 0.83) |
| 8 | Hydroxychloroquine use during follow-up | 0.79 (0.64 - 0.99) | 0.79 (0.64 - 0.99) | 0.63 (0.53 - 0.76) | 0.64 (0.53 - 0.76) |
| 9 | Sulfasalazine use during follow-up | 0.86 (0.75 - 1.00) | 0.86 (0.74 - 0.99) | 0.87 (0.64 - 1.17) | 0.87 (0.64 - 1.18) |
| 10 | Leflunomide use during follow-up | 1.54 (1.20 - 1.98) | 1.58 (1.24 - 2.01) | 0.88 (0.70 - 1.11) | 0.88 (0.70 - 1.11) |
| 11 | History of GC therapy three years before cohort entry | 1.20 (1.09 - 1.33) | 1.26 (1.15 - 1.39) | 0.87 (0.76 - 1.01) | 0.90 (0.78 - 1.03) |
| 12 | RA disease duration at cohort entry (log- transformed) | 0.98 (0.94 - 1.03) | 0.98 (0.94 - 1.03) | 0.93 (0.85 - 1.01) | 0.93 (0.86 - 1.01) |

* df = degrees of freedom

**Sensitivity analysis for Model 3 (current dose; 5mg/day)**

**Supplementary Table 3.** Sensitivity analyses using Model 3 in CPRD

|  | | HR (95% CI) |
| --- | --- | --- |
| 1 | Main analysis: Excluding patients with GC>40mg/day and all confounders^∂^ | 1.25 (1.19 - 1.31) |
| 2 | As analysis 1 but including patients with >40mg PED | 1.20 (1.17 - 1.24) |
| 3 | As analysis 1 but without family history of DM | 1.24 (1.19 - 1.31) |
| 4 | As analysis 1 but modelling GC use in 3 years prior to first RA code as cumulative dose rather than binary indicator | 1.26 (1.20 - 1.33) |
| 5 | As Analysis 4, adding BMI and smoking as additional confounders (with multiple imputation for missing values of BMI/smoking) | 1.14 (1.06 - 1.17) |

∂ All confounders refers to gender, age, squared term for age, family history of DM, history of hypertension, NSAIDs ever use at cohort entry, and concomitant time-varying use during follow-up of four main DMARDs, RA duration (log transformed) and prior GC use for the three years before cohort entry

**Supplementary Table 4.** Sensitivity analyses using Model 3 in NDB

|  | | HR (95% CI) |
| --- | --- | --- |
| 1 | Main analysis: excluding patients with GC>40mg/day and with confounders comparable to CPRD^∂^ | 1.30 (1.21 - 1.38) |
| 2 | As analysis 1, with additional confounders^¥^ | 1.18 (1.10 - 1.26) |

∂ Confounders comparable to CPRD refers to gender, age, history of hypertension and NSAIDs ever use at cohort entry, and concomitant time-varying use during follow-up of four main DMARDs, RA duration and GC use 3 years prior to cohort entry.

¥ Ethnicity, ever smoking status, BMI, employment status, rheumatic disease comorbidity index, total annual income, other DMARDs, biologic use and measures of disease severity (health assessment questionnaire (HAQ), pain scale, global severity scale).

**References**

1. Thomas SL, Edwards CJ, Smeeth L, Cooper C, Hall AJ. How accurate are diagnoses for rheumatoid arthritis and juvenile idiopathic arthritis in the general practice research database? *Arthritis and rheumatism* 2008;59(9):1314-21.
